# Circulating Fatty Objects and Their Preferential Presence in Pancreatic Cancer Patient Blood Samples

**DOI:** 10.3389/fphys.2022.827531

**Published:** 2022-02-14

**Authors:** Ruoxiang Wang, Nicholas N. Nissen, Yi Zhang, Chen Shao, Chia-Yi Chu, Carissa Huynh, Edwin M. Posadas, James S. Tomlinson, Michael S. Lewis, Stephen J. Pandol

**Affiliations:** ^1^Department of Medicine, Cedars-Sinai Medical Center, Los Angeles, CA, United States; ^2^Department of Surgery, Cedars-Sinai Medical Center, Los Angeles, CA, United States; ^3^Department of Biomedical Sciences, Cedars-Sinai Medical Center, Los Angeles, CA, United States; ^4^Biobank and Translational Research Core, Cedars-Sinai Medical Center, Los Angeles, CA, United States; ^5^Department of Surgery, VA Greater Los Angeles Healthcare System, Los Angeles, CA, United States; ^6^Department of Pathology, VA Greater Los Angeles Healthcare System, Los Angeles, CA, United States

**Keywords:** pancreatic cancer, pre-surgery cancer patient, blood sample, circulating fatty object, cancer-associated thrombosis, blood vessel occlusion, hyperthrombosis, biomarker

## Abstract

Human cancers are often complicated with increased incidences of blood vessel occlusion, which are mostly insensitive to anticoagulation therapy. We searched for causal factors of cancer-associated embolism. A total of 2,017 blood samples was examined for visible abnormalities. Examined were peripheral blood samples from cancer patients who were about to undergo surgical treatment for genitourinary, breast, gastrointestinal or abdominal tumors. Samples from ambulatory patients being treated for recurrent or castration-resistant prostate cancers were included in the study. The lipid-rich nature was studied with lipophilic stains and lipid panel analysis, while surface membrane was assessed with specific staining and antibody detection. We identified a new entity, lipid droplet-like objects or circulating fatty objects (CFOs), visible in the blood samples of many cancer patients, with the potential of causing embolism. CFOs were defined as lipid-rich objects with a membrane, capable of gaining in volume through interaction with peripheral blood mononuclear cells in *ex vivo* culture. Blood samples from pancreatic cancer patients were found to have the highest CFO incidence and largest CFO numbers. Most noticeably, CFOs from many pancreatic cancer samples presented as large clusters entangled in insoluble fiber networks, suggestive of intravascular clotting. This study identifies CFO as an abnormal entity in cancer patient blood, and a contributory factor to intravascular embolism during cancer development and progression.

## Introduction

Human cancers are associated with frequent intravascular embolism (Noble and Pasi, 2010), which could be either symptomatic or asymptomatic, occurring before or after the cancer diagnosis (Viale, 2005; Elyamany et al., 2014). Pancreatic cancer is found to have the highest incidence; from 17 to 57% of the patients suffering from embolic events (Khorana and Fine, 2004; Shah and Saif, 2010). As a cancer complication, the embolism is often fatal, accounted for the second leading cause of cancer mortality (Pruemer, 2005; Sheth et al., 2017). Intriguingly, major anticoagulation strategies (factor Xa inhibition, vitamin K antagonist and antithrombin activation) have only limited efficacies, while the embolism may recur regardless of the treatment. Delineating the mechanism of vascular embolism in cancer progression and metastasis will help improve patient survival. The aim of this study is to search for additional abnormalities which may contribute to cancer-associated vascular embolism.

Vascular embolism in clinical cancers has been studied extensively by the name of cancer-associated thrombosis. The most striking features include the haphazard vasculature infliction and general ischemic nature (Caine et al., 2002; Lee et al., 2017). The embolism may take place in both arterial and venous systems, suggesting that the main abnormality is in the blood rather than the vasculature. Embolism in the arterial system leads to ischemic vascular diseases like stroke, myocardial infarction, and other tissue and organ occlusions. Venous obstruction in superficial or deep veins may lead to pulmonary embolism. Intriguingly, pancreatic cancer is found to have high complications of stroke (Lee et al., 2017; Chan et al., 2018), heart attack (Takeshita et al., 2018; Yu et al., 2019), superficial or deep vein thrombosis (Horsted et al., 2012; Campello et al., 2019), and pulmonary embolism (Shen and Pollak, 1980; Ogren et al., 2006). Blood samples from clinical pancreatic cancer patients may be highly pertinent subjects for studying cancer-associated vascular embolism.

The causes of embolism during cancer progression and metastasis are multifaceted (Lip et al., 2002; Viale, 2005). Various risk factors may contribute to cancer patient blood occlusion. Visceral adenocarcinoma tumors produce mucins, which could be responsible for vascular lumen occlusion (Pinzon et al., 1986; Amico et al., 1989). Tumor growth and invasion may impair vascular integrity and activate the coagulation cascade, while intravascular tumor extension or circulating tumor cells may cause vascular embolization. Tumor cells and the surrounding reactive stroma may abnormally express pro-coagulant proteins such as the tissue factor, podoplanin, plasminogen activator inhibitor-1, and cancer procoagulant, raising the patient blood to a hypercoagulability or a hyperthrombolic state (Khorana et al., 2007; Abdol Razak et al., 2018). Furthermore, tumor cells may promote hemostasis by releasing metabolic intermediates, exosomes, extracellular matrices, and inflammatory cytokines (Elyamany et al., 2014; Falanga et al., 2017; Abdol Razak et al., 2018). Clinically, the embolism during cancer is difficult to manage with anticoagulation therapy; and in many cases anticoagulation therapies could not prevent its recurrence (Prandoni et al., 2002; Carrier et al., 2013; Timp et al., 2013; Khan et al., 2019). In fact, the risk of cancer-associated vascular embolism is not reduced in thrombocytopenia (Samuelson Bannow et al., 2018). These observations suggest that, besides hyperthrombosis, cancer patient blood has additional abnormalities that may contribute to the embolism.

For this report, we examined patient blood samples including those from pancreatic cancer patients for additional abnormalities that could be explored as etiologic factors or warning biomarkers of cancer-associated vascular embolism. Some of the samples contained large lipid droplet-like objects, which we named circulating fatty objects (CFOs), a previously unrecognized entity. CFOs are large, with the potential danger of acting as emboli. The incidence of CFOs in human cancers, together with CFOs’ differential appearance and peculiar structure in pancreatic cancer blood samples, implicated CFOs as important participants in cancer-associated vascular embolism.

## Materials and Methods

### Study Subjects

This study used a total of 2,017 human blood samples (see Table 1 for detailed sample information). Majority of the samples came from cancer patients just before surgery, most of which were for curative intent. Information on each sample’s organ of surgery were based on annotation records of the Biobank Laboratory. For comparative analysis, the samples were categorized into groups based on anatomical organs and systems of the disease. For the gastrointestinal surgery group, samples were obtained from 676 patients before surgical treatment of pancreatic cancer, and other gastrointestinal and abdominal diseases from September 10, 2013 to May 6, 2019. For the genitourinary surgery group, samples were obtained from 675 patients before surgical treatment from June 10, 2011 to March 15, 2019. For the breast cancer group, samples were obtained from 141 patients before surgical treatment of mammary gland diseases from August 11, 2015 to January 22, 2019. For the metastatic castration-resistant prostate cancer (mCRPC) group, 433 samples were obtained from 148 patients who visited Cedars-Sinai Medical Center for treatment of recurrent or mCRPC between April 25, 2011 and January 13, 2016. Many of the samples in this group were obtained by sequential sampling of the same patients at multiple returning visits during the period. For the control group, 74 of the 80 samples were obtained from healthy donors and another 6 were purchased from Zen-Bio (Research Triangle Park, NC).

**TABLE 1:** CFO incidence in different organs of cancer surgery.

Organ of surgery for the samples^a^	Number of samples	Number of samples with CFOs	Incidence rate^b^	Sample information—Organs of surgery and surgical procedures (Number of samples)^a^
Health donor	80	0		
mCRPC	433	14	3.23%	Ambulatory prostate cancer patients visiting oncologist for non-surgical treatment.
Adrenal gland	15	1		Adrenal tumor (6), and adrenalectomy (9)
Kidney	262	23	8.61%	Kidney (142), kidney cancer (2), nephrectomy (16), partial nephrectomy (70), radical nephrectomy (23), nephrourectomy (2), nephroureterectomy (4), renal cyst (2), and polycystic kidneys (1)
Prostate	332	40	12.05%	Prostate (189), prostate cancer (122), and prostatectomy (21)
Urinary bladder	65	2	3.08%	Bladder (43), urothelial carcinoma (1), cystectomy (8), complete cystectomy (1), and cystoprostatectomy (12)
Testicle	7	1		Testicle (2), testicular cancer (2), orchiectomy (2), and vesiculectomy (1)
Breast	141	13	9.22%	Breast (35), lumpectomy (19), mastectomy (58), segmental mastectomy (20), simple mastectomy (4), total mastectomy (1), axillary mass excision (1), chest wall mass excision (1), sternal wound resection (1), and breast reduction (1)
Pancreas	458	95	20.74%	Pancreas (181), pancreatic cancer (6), pancreatic mass (5), pancreatic resection (24), pancreatectomy (70), Whipple (167), pancreatic cyst (5)
Liver	179	20	11.17%	Liver (55), hepatic resection (82), radio frequency ablation (19), hepaticojejunostomy (5), hepatic lobectomy (5), liver biopsy (4), liver cyst (7), and laparotomy (2)
Gall bladder	10	2		Gall bladder (3), gall bladder cancer (3), cholecystectomy (1), cholecystectomy/mastectomy (1), ampullary cancer (1), and choledochal cyst (1)
GI tract	15	2		Gastric resection (1), duodenum (1), duodenal mass (1), duodenectomy (3), small bowel resection (1), colon (1), colon cancer (1), colon resection (5), and colon/hepatic resection (1)
Abdomen	20	1		Retroperitoneal tumor (3), abdominal mass excision (7), mesenteric tumor (3), metastatic nodule (1), abdominal washout (1), pelvic mass excision (1), bilateral inguinal lymph node excision (1), abdominal cyst (1), splenectomy (1), ovary (1)
Total	2017	214	10.61%	

*^a^For each sample, organ of surgery and surgical procedure information were based on annotation record of the Biobank.*

*^b^Only meaningful incidence rates (organs with large sample numbers) were calculated.*

All the samples were collected from median cephalic/cubital veins, and into the 10 ml lavender-top Vacutainertubes (BD366643, **Becton, Dickinson and Company,** Franklin Lakes, NJ), with dipotassium ethylenediamine tetra acetic acid (K_2_-EDTA) as an anticoagulant. The use of human samples for research was approved by the Institutional Review Board (IRB numbers Pro 00025217 and Pro 00030418). Informed written consent was obtained for the use of blood samples in research.

### Blood Sample Processing

The samples were transported to the Biobank Laboratory, where plasma from the samples was removed after centrifugation for clinical diagnosis and banking, leaving part of the buffy coat on top of packed red blood cells, which ranged from 0.1 to 3 ml in volume for research use. For this study, samples at this state were named as packed blood cells.

On arrival at the research laboratory, packed blood cell samples were diluted with a balanced salt solution containing 0.01% glucose, 5 μM CaCl_2_, 9.8 μM MgCl_2_, 540 μM KCl, 126 mM NaCl, and 14.5 mM Tris, pH 7.6 as we reported (Shao et al., 2014). For red blood cell removal, the sample was diluted by onefold. For microscopic examination of the sample, 0.1 ml packed blood cells were mixed with 1.9 ml dilution buffer to produce a 20-fold dilution. In some cases, 200-fold dilution was made for better examination.

Red blood cells were removed by ammonium chloride hemolysis, with a sterile hemolysis buffer (150 mM NH_4_Cl, 15 mM Tris, pH7.4, and 0.1 mM EDTA). For each sample, every 2 ml of 1:1 diluted packed blood cells were mixed with 8 ml of the hemolysis buffer. The sample was incubated at room temperature until complete hemolysis, which took place usually within 5 min. The sample was subjected to centrifugation at 300 × g for 10 min to recover the pellet, which was washed twice in phosphate buffered saline (PBS) and recovered in a pellet once more.

### Mixed *ex vivo* Culture and Circulating Fatty Object Enumeration

The recovered pellet was resuspended in 5 ml of T-medium (Formula LS0020056DJ, Thermo Fisher Scientific, Carlsbad, CA) containing 10% fetal bovine serum (FBS, Atlanta Biologicals, Lawrenceville, GA), penicillin (100 U/ml) and streptomycin (100 μg/ml). After being counted on a TC20 automated cell counter (BioRad, Hercules, CA) under trypan blue exclusion conditions, viable PBMCs were adjusted to 1 × 10^6^/ml so samples had the same initial cell numbers in *ex vivo* culture in 10-cm dishes (1 × 10^7^/dish). In patient samples with very low PBMC counts, all the isolated cells were plated. Immediately after plating, the number of CFOs mixed in the PBMCs was estimated based on counting under a microscope at low magnification (20×); and was rounded to the nearest 50 or 100. Mixed CFOs and PBMCs were then subjected to cell culture at 37°C in humidified atmospheric air supplemented with 5% CO_2_ for 8 weeks.

### Sample Staining

We previously reported our Oil Red O staining protocol (Zhau et al., 2011). Contrast dyes for fluorescence staining included CellMask green (C37608) and CellMask orange (C10045) plasma membrane stains, MitoTracker Green FM (M7514), LysoTracker Green DND-26 (L7526), CellTrace CFSE (C34570), and Hoechst 33342 (H1399, Thermo Fisher Scientific). CFOs in *ex vivo* culture were isolated from PBMCs with a pipet under a microscope and placed in 1 ml fresh culture medium in a plastic 12-well plate. In some cases, the samples were spiked with a small number of K562 cells (1 × 10^4^/ml) (ATCC, Manassas, VA) for control staining. Staining dyes were added to a final concentration of 2 μg/ml. Hoechst 33,342 was used to stain nuclei in live cells. After incubation at 37°C in dark for 10 min, stained samples were washed twice by gentle replacement of 80% of the staining medium with fresh medium without removing stained cells. Stained sample in fresh medium was then subjected to fluorescence microscopic inspection and imaging.

### Membrane Protein Detection

CFOs in the *ex vivo* culture were isolated with a pipet under a microscope and placed in 1 ml PBS. Biotinylated antibodies to perilipin (NB110-40760) or perilipin-2 (NBP2-23486B) were added to a final concentration of 10 μg/ml. Biotinylated IgG proteins (NBP1-97078 and NBP1-96846) from the same commercial source (Novus Biologicals, Centennial, CO) were used as respective isotype control. Incubated at room temperature for 2 h, samples were washed three times with gentle PBS changes. CFOs in 1 ml PBS were then incubated for 1 h with 2 μl of pre-cleared streptavidin conjugated Dynabeads from the Cellection Biotin Binder Kit (Thermo Fisher Scientific). After two gentle washes, CFOs in PBS were subjected to phase contrast microscope imaging.

### Lentiviral Infection

Lentiviral particles expressing a green fluorescence protein (GFP) (MISSION TurboGFP, Sigma-Aldrich, St. Louis, MO) were used to infect cell cultures with the manufacturer’s recommended protocol. Briefly, cell cultures in 0.5 ml culture medium in each well of a 6-well plate were infected with 10 μl (1 × 10^7^ TU) of viral particles. Polybrene (Santa Cruz Biotechnology, Dallas, TX) was used at 8 μg/ml to enhance infection efficiency. After 4 h of incubation at 37°C, 1.5 ml fresh medium was added, and the cultures were maintained for 4 weeks with daily fluorescence microscope inspection.

### Lipid Panel Analysis

CFOs in the *ex vivo* culture were isolated and placed in PBS for washing to remove contaminating PBMCs. Subsequently, 200 individual CFOs from each culture were loaded onto sample collection cards for determination of cholesterol, triglycerides, and high-density lipoprotein (HDL) (COREMEDICA Laboratories, Lees Summit, MO). To simplify the estimation of lipid concentration, all CFOs were assumed to be perfect spheres with a uniform diameter of 200 μm. Based on these assumptions, the total volume of the 200 CFOs in each sample would be 0.84 μl. Further assuming a 100 μl sample volume for lipid panel analysis, we calculated the lipid concentration inside CFOs by multiplying values from the lipid panel analysis by a factor of 120.

### Microscopic Documentation

Color photographs were obtained with an EVOS XL Core inverted microscope (Thermo Fisher Scientific). All other live cell phase contrast and fluorescence images were taken with an Eclipse Ti inverted microscope (Nikon Instruments Inc., Melville, NY), as we reported (Wang et al., 2019).

## Results

The aggressive behavior and high lethality of cancer-associated vascular embolism before and after cancer diagnosis suggest that, many cancers may have already become a systemic disease at the time of diagnosis (Perkhofer et al., 2014; Sohal et al., 2014). Suspecting additional contributory factors of vascular occlusion, we examined whether certain features in cancer patient peripheral blood samples could serve as sentinels of cancer-associated vascular embolism.

### Circulating Fatty Objects in Cancer Patient Blood Samples

**FIGURE 1:**
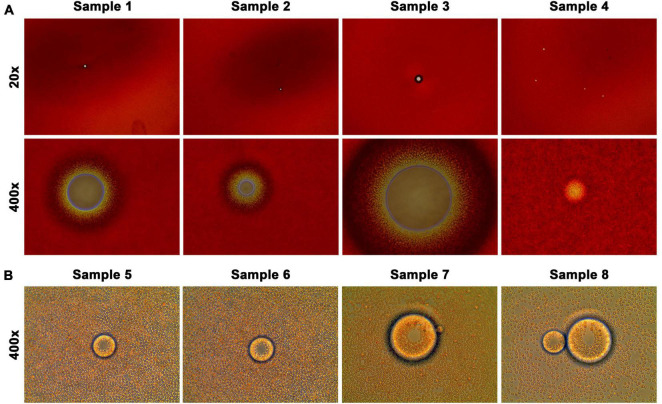
CFOs in clinical cancer patient blood samples. Packed blood cells from clinical cancer patients were diluted and spread into a thin layer for CFO observation under an inverted microscope. Representative results are shown. **(A)** When packed blood cells were subjected to 20-fold dilution, CFOs in samples could be seen as transparent dots against a thick background. CFOs can be detected at 20× magnification (upper row). In sample 4, 4 small CFOs could be seen. At 400× magnification (lower row), the relationship between CFOs and red blood cells can be appreciated. **(B)** When packed blood cells were subjected to 200-fold dilution, CFOs became prominent among the red blood cells.

We used packed blood cells as starting materials to examine cancer patient blood samples, after the plasma was harvested by clinical laboratory. On arrival at the research laboratory and before isolating PBMCs by density gradient centrifugation (Shao et al., 2014), a balanced salt solution was used to dilute packed blood cells. During this process, we found unexpectedly that some patient blood samples contained a peculiar type of previously unrecognized object. When a thin membrane of diluted blood cells was examined under a microscope at low magnification (20× or 40×), these objects were bright ghost spots on a background of red blood cells (Figure 1A). Further dilution of the blood cells in combination with careful microscopic examination revealed the objects’ shape and size relative to the surrounding red blood cells (Figure 1B).

These objects appeared as almost perfect spheres with adhering red blood cells. Using red blood cells as scale, 7 μm in diameter, we estimated that these objects had dimensions ranging from 100 to 1,000 μm or even larger in diameter (Figure 1). Some of the objects could indeed be seen with the naked eye. On the other hand, these objects could be mechanically disintegrated (e.g., by pipetting) into smaller sizes below 100 μm in diameter. The smaller objects remained as spheres and never dissolved or disappeared into the aqueous phase, suggesting a lipid-rich nature. Until components of the subject are fully determined, we provisionally named the newly recognized entity CFO.

### Circulating Fatty Object Lipid Characteristics

To examine CFOs in detail, we used ammonium chloride hemolysis to remove red blood cells, leaving CFOs mixed with PBMCs. CFOs were picked from the mixture with a pipet tip for further examination. We first based on morphologic and behavioral inspection together with negative Hoechst 33,342, MitoTracker and LysoTracker stain to determine that CFOs were not living cells; and contained no cell nucleus nor subcellular organelles. We then employed several methods to investigate the lipid nature of CFOs.

**FIGURE 2:**
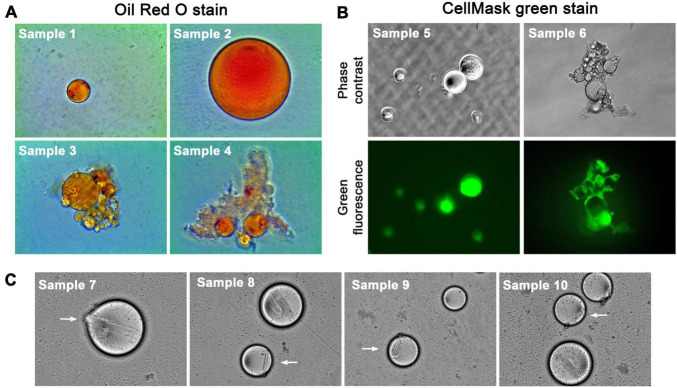
CFOs have a lipid-rich content. Representative images are shown. **(A)** CFOs isolated from 4 samples of pancreatic cancer were stained with Oil Red O. Red color represents positive staining. All images were taken at 100 × magnification with the same microscope. Note the clustered CFOs entangled in fiber networks in Samples 3 and 4. **(B)** Representative results of the CellMask green stain of 8 CFO samples from pancreatic cancer (100×). Sample 5 represents the 6 cases in which CFO contents were stained after 30 min. Sample 6 represents the 2 cases in which only the CFO surface was stained after 30 min. Note bundled CFOs entangled with fiber networks in Sample 6. **(C)** Cholesterol-like crystals in CFOs are indicated with arrows (100×).

First, CFOs from 4 patients were fixed with formalin and subjected to Oil Red O staining. CFOs showed red staining in all the CFOs tested (Figure 2A). Second, when stained with plasma membrane specific dyes of CellMask green or CellMask orange, many CFOs were stained entirely rather than limited on the surface. For instance, in a study where 8 patient samples were tested, some CFOs in each sample were stained entirely while other CFOs were stained only on the surface (Figure 2B); and the stain pattern was not changed by extended staining time from 10 min to 2 h. CellMask dyes contain lipophilic moieties for plasma membrane integration. Individual CFOs with positive staining may have a higher lipid composition, while presence of CFOs with surface-restricted stain may be suggestive of heterogeneity of the CFO population. Third, many CFOs contained crystal-like structures (Figure 2C), similar to the typical plate-like monohydrate cholesterol crystals in gall bile (Portincasa et al., 1996, 1997). Finally, 6 CFO samples were subjected to lipid panel analysis. For each sample, 200 CFOs were picked from the first week of mixed *ex vivo* culture. The 200 CFOs had an estimated total volume of 0.84 μl. Using this estimate, values of the lipid panel analysis were deduced as high lipid concentrations (Table 2). These findings support the lipid-rich nature of most CFOs.

**TABLE 2:** Calculated cholesterol and triglyceride concentrations in 6 CFO samples.

CFO sample	Sample 1	Sample 2	Sample 3	Sample 4	Sample 5	Sample 6
Lipid profile service ID	10146404	10146405	10146406	10146407	10146408	10146409
Cholesterol (mg/dL of blood)^a^	75	70	68	72	72	83
Cholesterol (mg/dL of CFO)^b^	9,000	8,400	8,160	8,640	8,640	9,960
Triglycerides (mg/dL of blood)^a^	34.1	37.7	43.7	46.3	45.2	41.4
Triglycerides (mg/dL of CFO)^b^	4,092	4,524	4,884	5,556	5,424	4,968
HDL (mg/dL of blood)^a^	11	12	11	10	10	10
HDL (mg/dL of CFO)^b^	1,320	1,440	1,320	1,200	1,200	1,200

*^a^Values obtained from lipid profile service with assumption of 100 μl plasma is used in test.*

*^b^Values calculated from a sample of 200 CFOs each with a diameter of 200 μm.*

To investigate whether CFOs were due to severely elevated blood lipids, we searched for CFOs in blood samples that contained lipemic plasma. In the 80 donor blood samples, 9 samples were found with lipemic plasma, while none contained any CFOs. In the 433 samples of mCRPC patients, none of the 14 samples containing CFOs was lipemic. The appearance of CFOs was, therefore, not associated with lipemia.

Paradoxically, CFOs always settled to the bottom of the diluted blood preparation and the *ex vivo* culture, never floating to the surface. When CFO-containing blood samples were subjected to Ficoll-Paque PLUS (density 1.077 g/ml, GE Life Sciences) or Ficoll-Paque PREMIUM (1.084 g/ml, Sigma-Aldrich) density gradient centrifugation, at least some of the CFOs were found in the red blood cell fraction, which has a density of 1.110 g/ml (Norouzi et al., 2017). These observations suggest that CFOs contained not only lipids but also other dense components.

Majority of the packed blood cell samples in this study were derived from preoperative patients who were to undergo cancer surgery, for which Propofol was a common anesthetic medication. Propofol was formulated in an Intralipid-like emulsion containing soybean oil, which had a density of 0.91 g/ml around 37°C (Noureddini et al., 1992; Sahasrabudhe et al., 2017). Though most blood samples were drawn when patients were conscious before anesthesia induction, we nevertheless investigated whether CFOs in patient blood could be due to Propofol use. Since Intralipid emulsion (Sigma-Aldrich) had almost the same formulation as Propofol emulsion, we simulated Propofol infusion by incubating 12 blood samples with Intralipid to detect for CFO formation. Freshly acquired donor whole blood samples were incubated with Intralipid, which was added to the blood in 0.5, 1, 5, and 10% (v/v) concentrations. The samples incubated at 37°C with rotary mixing at 72 rpm/min for 2 h. Subsequently, the samples were subjected to centrifugation at 300 × g at 4°C for 10 min to remove the plasma fraction. Removed was also the Propofol emulsion, which was seen in upper part of the plasma fraction. Packed blood cells were then processed for red blood cell removal and examination of any CFO formation. None of the samples produced any CFOs after incubation for 2 h. Extended overnight incubation did not lead to CFO formation either. It is most likely that CFOs are a bona fide new entity in circulating cancer patient blood.

### Circulating Fatty Object Surface Membrane

**FIGURE 3:**
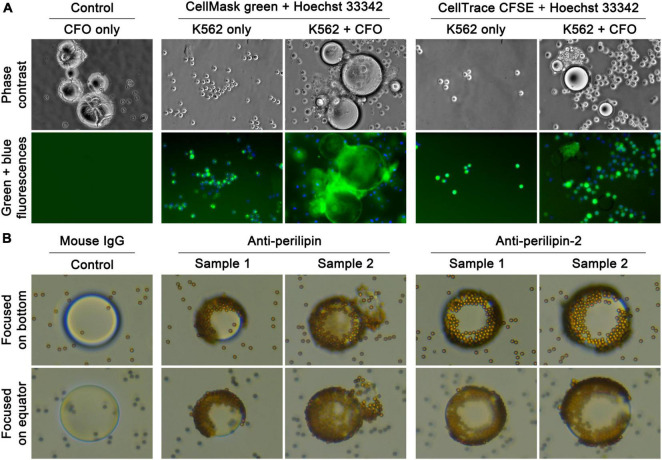
Basic CFO structure. Basic CFO structure was investigated by determining the presence of an enveloping surface membrane and absence of a cell nucleus-like structure. **(A)** Some CFOs displayed a wrinkled surface (Control, CFO only) suggesting the presence of an enveloping membrane. The enveloping surface membrane and absence of a nucleus-like structure were investigated with positive staining by membrane-specific CellMask green and negative staining of DNA-binding Hoechst 33,342, respectively. K562 cells were added to indicate positive staining (200×). CellTrace CFSE failed completely to stain CFOs. **(B)** An indirect detection method for CFO membrane was tested by detecting membrane perilipin and perilipin-2 proteins with biotin-conjugated specific antibodies, which were revealed by secondary binding of streptavidin-conjugated nanoparticles, each of which was 4.5 μm in diameter. During microscopic imaging (400×), when the focus was set on bottom of a CFO, nanoparticles binding specifically on the CFO could be seen.

Microscopic examination suggested that CFOs had an enveloping membrane. In many cases, CFOs showed a wrinkled surface on isolation (Figure 3A). In other cases, CFO surface at isolation had many adherent PBMCs, which were not seen to penetrate to the CFO proper, suggesting the presence of a membranous barrier. When membrane specific CellMask green or CellMask orange was used as stain, many CFOs from the 8 packed blood samples were seen with circular profiles of the surface (Figure 3A). In comparison, when samples were stained with CellTrace CFSE, which would penetrate plasma membrane to stain cytosolic free amines, CFOs were completely negative in contrast to the control K562 cells (Figure 3A), suggesting that CFOs contained little protein.

In complementary studies, we developed an indirect assay to detect CFO membrane proteins. Biotin-conjugated antibodies to perilipin or perilipin-2 were used to stain CFOs, and the stain was visualized with streptavidin-conjugated Dynabeads, each of which was 4.5 μm in diameter. Compared to the IgG isotype control group, large numbers of Dynabeads were seen adhered to the CFO surface (Figure 3B), suggestive of the presence of perilipin family lipid droplet proteins. These studies suggested that the lipid-rich CFO was composed of a phospholipid membrane, which is decorated specifically with the perilipin family of lipid droplet proteins (Sztalryd and Brasaemle, 2017; Exner et al., 2019).

### Circulating Fatty Objects Interacted With PBMCs to Gain in Size

**FIGURE 4:**
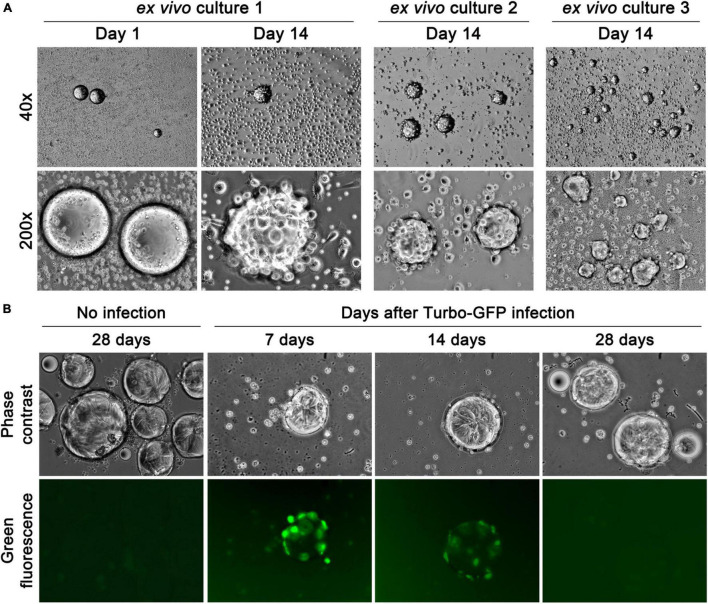
CFOs gain in volume by annexing PBMC blasts. Representative images of mixed *ex vivo* culture of CFO and PBMCs are shown. **(A)** In *ex vivo* culture 1, images show PBMCs on the CFO surface at the beginning of the culture (Day 1), and PBMC blast interaction with CFOs in 2 weeks (Day 14). In *ex vivo* cultures 2 and 3, two additional examples of the interaction are shown. **(B)** Tracking the fate of PBMCs fused to CFOs with GFP reporter expression (100×). After hemolytic isolation, samples were placed in mixed *ex vivo* culture and infected with MISSION TurboGFP lentiviral particles on the first day of culture (Day 1, not shown). PBMC fluorescence was tracked for 4 weeks.

In packed blood cell samples, CFOs often had many adherent blood cells (Figure 1). We assessed the fate of CFOs among PBMCs by observing their interaction. From individual patient blood samples, CFOs mixed with PBMCs were subjected to *ex vivo* culture for 8 weeks. CFOs in culture had adherent PBMCs (Figure 4A). For many samples, PBMCs rarely became activated to grow in *ex vivo* culture; and they would die in the first 2 weeks, as we reported (Wang et al., 2016). In most of these cultures, CFOs remained with sustained morphology and numbers for the entire 8 weeks. In contrast, the volume of CFOs did increase in some mixed *ex vivo* cultures. Daily inspection revealed that many PBMCs in these cultures could become blasts, activated to grow larger in size with macrophage or dendritic cell morphology (Shaqinah et al., 2016), while PBMC blasts on CFO surfaces could fuse with the CFO (Figure 4A). As culture proceeded, PBMC blasts fused into CFOs would disappear, resulting in an enlarged CFO sphere. After 8 weeks of culture, Hoechst 33,342 stain was used to determine that these CFOs did not retain detectable genomic materials of the PBMCs. Staining with MitoTracker or LysoTracker did not detect any signs of mitochondria or lysosomes either, indicating a complete resolution of the PBMC blasts.

In complementary studies, we tracked the fate of the CFO-adhering PBMCs by GFP expression. At the beginning of the culture, 8 samples with CFOs mixed in PBMCs were infected with lentiviral particles of MISSION TurboGFP. Green fluorescence was emitted only from PBMCs, not from CFOs, confirming that the latter lacked gene expressional capability. In 3 of the 8 samples, PBMCs underwent blast later, emitting green fluorescence on CFO surface and after fusion to the CFO (Figure 4B). The fluorescence, however, would disappear gradually in 4 weeks, indicating mortality and disintegration of the PBMC blasts. These results indicated that CFOs could gain volume through interacting with certain blood cells.

### Circulating Fatty Objects Clusters in Pancreatic Surgery Samples

By examining the presence of CFOs in a total of 2,017 blood samples, we found that CFOs appeared differentially in different cancer groups. No CFOs were detected from the group of 80 healthy donor samples. In the mCRPC group, 14 of 433 samples were found to contain CFOs (3.23%). In the genitourinary surgery group, 67 of the 675 samples were found to contain CFOs (9.93%). In the breast cancer group, 13 of the 141 samples contained CFOs (9.22%). In the gastrointestinal surgery group, 119 of the 684 samples were found with CFOs (17.40%). Gastrointestinal surgery patients had the highest incidence of CFOs in their blood samples.

We further analyzed CFO incidence by the organ of surgery (Table 1). This analysis revealed that blood samples from pancreatic cancer surgery had the highest CFO incidence (20.43%), followed by prostate (12.42%), liver (11.17%), breast (9.22%), and kidney cancer (8.61%). In comparison, only 2 samples from the 64 urinary bladder cancer surgeries were found to contain CFOs.

**FIGURE 5:**
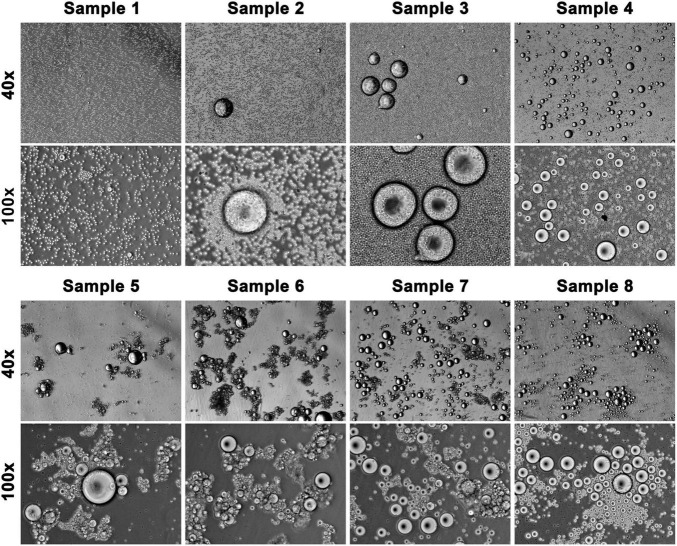
CFOs in pancreatic cancer patient blood samples. Packed blood cells from pancreatic cancer patients were subjected to hemolysis. Recovered CFOs mixed in PBMCs were subjected to *ex vivo* culture. CFOs in 8 representative cultures are shown. All photos were taken on the first day of culture. In the upper panels, CFOs are seen among a background of PBMCs (which have a diameter of 10 μm). In the lower panels, the samples had more CFOs than PBMCs. Many of these samples (e.g., Samples 5, 6, and 7) contained mainly CFO clusters entangled with fiber networks.

**FIGURE 6:**
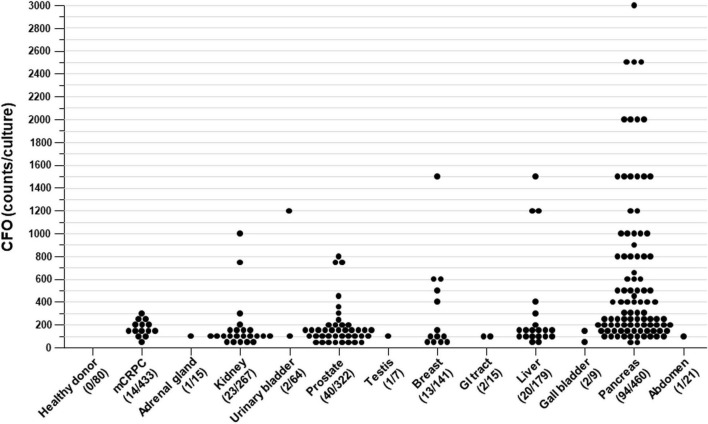
CFO incidence based on cancer type. Each data point represents estimated CFO numbers in a mixed *ex vivo* culture. Data points were plotted based on the patient’s cancer type.

In addition to the high incidence, CFOs from the pancreatic cancer surgery group displayed unique properties. While all the CFOs shared common features as described in the preceding sections, CFOs from certain pancreatic cancer samples displayed these features in a drastic fashion. Many samples from pancreatic cancer contained large numbers of CFOs, which were even higher than PBMC counts in some cases, as few PBMCs were recovered from these packed blood cell samples in the first place (Figure 5). Critically, these CFOs were often present in bundled clusters, entangled in fiber networks when isolated and impossible to be fully resuspended in culture medium (Figure 5). With too few PBMCs, these clusters stayed docile in culture, tangled in their networks for 8 weeks without any signs of network disintegration. The differential CFO occurrence in pancreatic cancer blood could also be seen when estimated CFO counts were compared (Figure 6). When CFO incidence in pancreatic cancers was compared based on surgical procedures (partial pancreatectomy, pancreatectomy, and Whipple), no significant difference was found. Additional study is needed to assess the relationship between CFO count and the status of cancer progression.

## Discussion

By examining packed blood cell samples of cancer patients, we unexpectedly identified CFOs as an abnormal entity (Figures 1A,B). Without literature resources for help, we will discuss clinical significance of the study by contemplating several intriguing aspects of the laboratory finding.

### Circulating Fatty Objects as Insoluble Emboli

CFOs displayed lipid properties. They were insoluble in PBS or cell culture medium. In addition, most CFOs were positive to lipophilic Oil Red O dye stain (Figure 2A), and many showed rapid internalization of the lipophilic CellMask green dye (Figure 2B) and internal structures similar to cholesterol crystals (Figure 2C). Estimated results based on lipid panel analyses validated CFO lipid constituents (Table 2). CFOs have a membrane, which was detected with cytoplasmic membrane specific CellMask green stain (Figure 3A). We designed a Dynabead detection method to indicate the presence of perilipin family proteins decorating the phospholipid membranes (Figure 3B). Our primary characterization thus defined the CFO as a lipid-rich droplet with a perilipin-decorated membrane.

Though the size of CFOs was seen in the range of 100–1,000 μm in diameter, actual size of CFOs in the circulation *in vivo* could be larger. In this study, CFOs were characterized *in vitro*, after blood samples were processed with mechanical forces of shearing, centrifugation, and resuspension, which could break CFOs. As a physical entity, CFO can act as an embolus, an underlying mechanism for the high recurrent vascular occlusion disregarding anticoagulation therapy.

### Circulating Fatty Objects and Hemostasis

Another important characteristic of CFOs was the formation of large clusters entangled with fiber networks (Figure 5). Collected directly in K_2_-EDTA Vacutainers and transported in ice, none of the blood samples used in this study was clotted on arrival at our laboratory. Though identity of the network proteins has yet to be determined, the appearance of these networks, and the formation of CFO clusters entangled with the networks likely took place in the patient’s circulation prior to blood draw. With their high stability as observed in the 8 weeks of mixed *ex vivo* culture, the pathologic role of CFO clusters in cancer-associated vascular embolism should be critically evaluated, as irregular objects in blood circulation may elicit hemostatic reaction.

Isolated from blood samples, the fiber networks were most likely derived from fibrin. In our experimental setting, CFOs and entangled fiber materials were studied in suspension in cell culture medium or buffered solutions, making it difficult to use immunostaining to identify nature of the fiber proteins. Corresponding experimental procedures must be designed to address this technical issue. Thrombogenicity testing will be conducted in the future to assess involvement of CFOs in the process of thrombosis.

### Circulating Fatty Object and Pancreatic Cancer Lethality

The packed blood samples used in this study were derived from patients with different cancer types (Table 1), each of which was treated with different surgeries. We examined CFO incidence by the patients’ organ of cancers. The analysis identified pancreatic cancer as having the highest CFO incidence (Table 1 and Figure 6). The trend of CFO incidence is similar to the clinically observed trend of cancer-associated vascular embolism, in which pancreatic cancer patients were found to have the highest incidence of vascular embolism (Caine et al., 2002; Khorana and Fine, 2004; Shah and Saif, 2010).

Pancreatic cancer, mainly pancreatic ductal adenocarcinoma, is one of the most aggressive and least treatable human malignancies. Compared to cancers in other organs, cancer in the pancreas is often too concealed or latent for early detection. By the time of disease manifestation, only a small fraction of tumor cases remains surgically resectable, while tumors in most patients are already metastasized, especially to the liver (Peixoto et al., 2015; Houg and Bijlsma, 2018). Cancer of the head of the pancreas causes gallbladder damage and biliary obstruction. Though difficulty of detection is a contributing factor, the lethality of pancreatic cancer is not fully understood. Pancreatic cancer may have unique characteristics favoring fast progression and high lethality (Hruban et al., 2019). On the other hand, we found that CFOs in pancreatic cancer often formed large clusters with fiber networks, highly stable in *ex vivo* culture. It is probable that CFOs and the CFO clusters exacerbate pancreatic cancer mortality either by occluding blood flow or by eliciting the coagulation cascade to elicit clot formation. High CFO incidence may contribute to the high lethality of pancreatic cancer. The existence of large numbers of CFOs, particularly CFO clusters entangled with fiber networks in patient blood, should be regarded as an etiologic factor for the high incidence of vascular occlusion in pancreatic cancer.

### Dynamic Interaction Between Circulating Fatty Objects and PBMCs

We used mixed *ex vivo* culture to assess the possible fate of CFOs, which turned out to be affected by external conditions. CFOs remained stable for the entire 8 weeks of the study if the admixed PBMCs showed little growth. CFOs gained volume when a certain cell types of the PBMC compartment became activated to grow (Figure 4A) and were then annexed by the CFOs (Figure 4B). These PBMC blasts attached to culture ware tightly displaying dendritic cell morphology, indicating a monocyte/macrophage origin (Shaqinah et al., 2016). Detailed investigation of the interaction between CFOs and PBMCs will provide insights into the origin and dynamics of CFOs circulating in cancer patient blood.

### Circulating Fatty Object as a Missing Link Between Gallbladder Diseases and Cardiovascular Incidents

Given that CFOs may grow through interaction with PBMC blasts, the origin of CFO is presently unknown. Since CFOs appeared more frequently in gastrointestinal cancers (Table 1 and Figure 6), we suspect two organs, the liver and gallbladder, as potential sites of CFO formation. The liver is important for lipid biosynthesis and metabolism, and a frequent site of cancer metastasis. Alternatively, CFOs may represent non-miscible compartment of the gall bile (Admirand and Small, 1968; Hofmann and Mysels, 1992), which is often leaked into blood circulation upon cancer-induced gallbladder damage. Intriguingly, not only gallbladder cancer but some non-malignant gallbladder diseases are associated with increased risk of vasculature occlusions (Wei et al., 2014; Chen et al., 2015), while cholecystectomy reduces the risk (Wei et al., 2019). Once proven with a gallbladder origin, CFO could be the missing link between gallbladder diseases and cardiovascular incidents. Further studies are warranted to identify the origin of CFO formation.

### Limitations of the Study

In the pancreatic cancer group, packed blood cell samples were derived from pancreatic cancer patients with resectable tumors prior to surgery. It would be intriguing to examine the presence and structural features of CFOs in blood samples of unresectable pancreatic cancers. Examination of blood samples from patients with cardiovascular incidents may also determine whether CFOs are related to non-cancer-associated vascular occlusions.

Due to the exploratory nature of this study, we used patient samples based on availability without specifying sample volume in the experimental setting. Samples of packed blood cells in this study ranged from 0.1 to 3 ml in volume. CFOs in some cancer patients might have not been detected due to limited sample volume. Future studies with sufficient sample volumes and pre-anesthesia blood draw will be carried out to validate the findings from this study.

Though commercial lipid panel analysis was used to estimate the amounts of lipids, detailed lipid components in CFOs are far from identified. Quantitative analysis with liquid chromatography and mass spectrometry may delineate the full profile of CFO components. Comparative analysis with liver fats and bile expiration may help identify the source of CFO formation.

## Data Availability Statement

Representative results are presented in the article. Further inquiries can be directed to the corresponding author/s.

## Ethics Statement

The studies involving human participants were reviewed and approved by the Institutional Review Board, Cedars-Sinai Medical Center. The patients/participants provided their written informed consent to participate in this study.

## Author Contributions

RW designed assays, conducted the experiments, performed the data analysis, and wrote the manuscript. YZ, CS, and C-YC conducted the experiments. NN, CH, EP, JT, and ML provided patient blood samples. SP coordinated and oversaw the project, and co-wrote the manuscript. All authors contributed to the article and approved the submitted version.

## Conflict of Interest

The authors declare that the research was conducted in the absence of any commercial or financial relationships that could be construed as a potential conflict of interest.

## Publisher’s Note

All claims expressed in this article are solely those of the authors and do not necessarily represent those of their affiliated organizations, or those of the publisher, the editors and the reviewers. Any product that may be evaluated in this article, or claim that may be made by its manufacturer, is not guaranteed or endorsed by the publisher.
